# Effects of Rapid Cooling on Properties of Aluminum-Steel Friction Stir Welded Joint

**DOI:** 10.3390/ma14040908

**Published:** 2021-02-14

**Authors:** Hamed Aghajani Derazkola, Eduardo García, Arameh Eyvazian, Mohammad Aberoumand

**Affiliations:** 1 Department of Mechanics, Design and Industrial Management, University of Deusto, 48007 Bilbao, Spain; e.garcia@deusto.es; 2 Department of Mechanical and Industrial Engineering, Qatar University, Doha 2713, Qatar; 3 School of Mechanical Engineering, College of Engineering, University of Tehran, Tehran 1417466191, Iran; m.aberoumand@ut.ac.ir

**Keywords:** cooling assisted friction stir welding, dissimilar materials, mechanical properties, microstructure evaluation

## Abstract

In this study, dissimilar sheets including AA3003 aluminum and A441 AISI steel were welded via cooling-assisted friction stir welding (FSW). Three different cooling mediums including forced CO_2_, forced water, and forced air were employed, and a non-cooled sample was processed to compare the cooling-assisted condition with the traditional FSW condition. The highest cooling rate belongs to CO_2_ and the lowest cooling rate belongs to the non-cooled sample as FSW. The best macrograph without any segregation at interface belongs to the water-cooled sample and the poorest joint with notable segregation belongs to the CO_2_ cooling FSW sample. The CO_2_ cooling FSW sample exhibits the smallest grain size due to the suppression of grain growth during dynamic recrystallization (DRX). The intermetallic compound (IMC) thickening was suppressed by a higher cooling rate in CO_2_ cooling sample and just Al-rich phase was formed in this joint. The lowest cooling rate in the FSW sample exhibits formation of the Fe rich phase. The IMC layers were thicker at the top of the weld due to closeness with the heat generation source. The water cooling sample exhibits the highest tensile strength due to proper mechanical bonding simultaneously with optimum IMC thickness to provide appropriate metallurgical bonding. Fractography observation indicates that there is a semi-ductile fracture in the water cooling sample and CO_2_ cooling sample exhibits more brittle fracture. Hardness evaluation reveals that the higher the cooling rate formed, the higher the hardness in stir zone, and hardness changes in the aluminum side were higher than the steel side.

## 1. Introduction

Al3xxx series is one of the aluminum alloys series that has more strength than the Al1xxx. Aluminum and manganese are the two main elements that make this alloy stronger than the Al1xxx series. High tensile strength, high plastic area, suitable weldability, high ductility, and proper corrosion resistance are some of this series’ features [1]. Decent ductility, fair strength, and being affordable are some of the features that make low carbon steels used extensively in the automotive industry [2,3,4].

In 1991 at TWI, friction stir welding (FSW), a solid-state joining process was invented [5,6,7,8]. High quality, efficiency, environmental protection, and energy-saving are guaranteed in this method [9]. The workpieces are intermixed mechanically by the traverse movement of the tool along the joint line, and mechanical pressure forms the smooth material [10,11]. At first, the FSW process was used for aluminum alloys [6,12,13,14]; however over time, the capacity and potential of FSW proved that this method could be employed not only for aluminum alloys, but also for other purposes such as joining of similar and dissimilar polymeric and metallic materials [15,16,17,18,19,20,21,22,23,24,25]. One of the useful structures that can be made by FSW is a steel-aluminum dissimilar joint [12,26,27]. This joint is a new class of dissimilar structure that can be used in various components in order to lighten up structures [13,28,29,30].

The majority of earlier research on high melting point materials focused on the interaction between the process parameters, the final weld microstructure, and the correlative mechanical features [31,32]. These studies and examinations have indicated that the cooling rate and peak temperature have significant effects on the joint mechanical features. For instance, the joint mechanical features will mostly depend on the final grain size, which is affected by heat input, whenever the welding peak temperature becomes lower than the phase transformation temperature [33]; while in different circumstances, when the welding peak temperature is higher than the phase transformation, the parameter which will play a significant role in the last phase composition and morphology is the cooling rate [34]. Furthermore, the grain structure before the phase transformation is very crucial [35]. Rapid cooling friction stir welding (RCFSW) implements a satisfying solution that synchronizes the cooling action and the welding process [36]. The same situation as the normal cooling process will be recreated in the case of an annealing action in a short amount of time, and a suitable temperature assumed for the cooled weld [37]. Investigating the microstructure evolution during the deformation stage and the cooling stage will be supported individually by this technique. There have been new studies on the effect of rapid cooling on FSW, such as the case study of Godasu et al. [36] which reported a significant advancement of 460 MPa in the yield strength of the joint with a very low reduction of ductility (3%), after cryocooling Al 5083 alloy to 90% thickness reduction. The main reason for this reduction is the existence of large defect volumes after the deformation, which causes premature plastic instability with no strain hardening. Zhang et al. [38] explored the effect of underwater FSW on the mechanical properties of the 2219Al–T6 joints and reached a maximum tensile strength of 360 MPa, which was higher than that of the typical FSW joint. In the investigation by Patel et al. [37] on cooling reinforced FSW to join Al–Cu by suggesting compressed air and water as cooling source (in the form of a jet) at the trailing side of the tool, considerable enhancement in tensile strength with the smoother stirred surface in case of CFSW of the water-cooling source was observed as compared to heating assisted FSW and normal FSW. Aghajani Derazkola and Khodabakhshi [39] studied the dissimilar bonding between Al–Mg aluminum alloy and A441 AISI steel by the UFSW process under different water medium temperatures. In their research, the room temperature water reported as the optimum cooling medium with a maximum tensile strength of ~310 MPa and elongation of ~13%. Kar et al. [40] applied liquid CO_2_ for joining similar AISI 1080 steel, wherein toughness and ductility were improved.

Among outstanding research about FSW of aluminum alloys and steels, the control of microstructure and optimization of intermetallic compound (IMC) thickness in these joints are not completely clear. A wide range of tests is needed to optimize IMC thickness and microstructure. In this research, we designated a typical 3xxx series Al alloy and 441AISI steel state as the target base material that can be used in automobile industries. On the other hand, to decrease the FSW test numbers (due to trying and error tests and using various process parameters) to form optimum IMC thickness and mechanical interlocking in the base metals interface, the extra cooling assists FSW was employed. In general, the aim of this article optimization of IMC thickness with optimization mechanical interlocking. These optimum parameters help decrease the number of experimental tests like examine various ranges of FSW tool rotational speed, traveling speed, tool tilt angle, tool offset, tool pin profile, and other parameters.

## 2. Experimental Procedure

**Figure 1:**
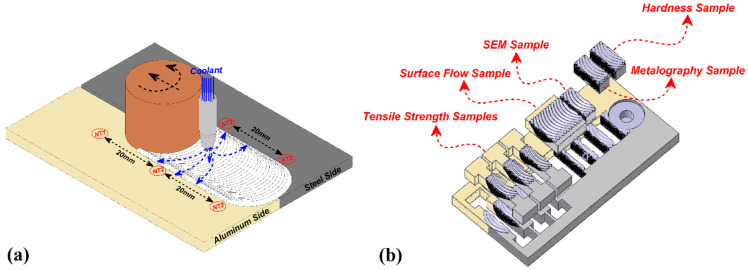
(**a**) Schematic of thermocouple places and (**b**) mechanical testing samples.

In this research, AA3003 aluminum alloy (Arak aluminum, Arak, Iran) and A441 AISI steel (Foolad Mobarakeh, Isfahan, Iran) sheet metals are welded with a FSW machine. The mechanical properties and chemical composition of base metals are presented in Table 1 and Table 2, respectively. The FSW machine in this study is a modified 3D axis milling machine (Machine Sazi Tabriz, Tabriz, Iran) with a welding fixture assembled on it. The interfaces of base metals were polished and smoothed, and the oxide was removed by sandpaper before welding procedure. In the experiment procedure design, the aluminum alloy (Al) was located on the retreading side (RS) and the steel (St) was placed on the advancing side (AS). The welding procedure was done by a tungsten carbide-made tool with frustum pin. The optimum process parameters were achieved after trial and error tests. The FSW process parameters are presented in Table 3. A 4 mm nozzle was used for the forced cooling rate. The nozzle was fixed 20 mm backside the tool and moved with the same direction and speed of the welding tool. In this research, air (air-forced cooling FSW (AF-FSW)), water (water-forced cooling FSW (WF-FSW)), and carbon dioxide (CO_2_) (CO_2_-forced cooling FSW (CF-FSW)) were used as coolants. Flow rates of coolants were the same: 120 mL/min, and during the exhaust from the nozzle, the coolants temperatures were 30 °C for air, 5 °C for water, and −35 °C for CO_2_, which were measured by laser thermometer during outlet. The flow rate was achieved by dividing total outlet volume of fluid by nozzle heat area per time period. During the FSW process, frictional heat and cooling rate were measured in the different conditions using three K-type thermocouples (Omega, Norwalk, USA). The schematic view of the FSW process and the thermocouple location are shown in Figure 1. The results of cooling-assisted FSW were compared with conventional FSW condition. For the mechanical behavior evaluation of the joints, tensile samples were prepared perpendicular to the weld line according to the ASTM/E8-M03 standard. Vickers micro-hardness was also measured along the joint to evaluate the base metal hardness after welding. Three specimens were cut by a wire electro-discharge machine, and the average results are presented. Metallographic observation was done by optical microscopy (OM) (Olympus Microscope, Tokyo, Japan). Average grain size was determined according to the ASTM E112-13 standard testing method. The OM was equipped with image processing software and average grain size was assessed and measured manually by using image processing software. The data was analyzed and plotted as a histogram in order to report relation between frequency of grain size. For investigation of microstructure and intermetallic layer in the weld line, scanning electron microscopy (SEM) (TESCAN Mira, Brno, Czech Republic) equipped with energy dispersive spectroscopy (EDS) (Bruker, Billerica, USA) with monochromatic Cu Ka radiation and continuous scan mode at 0.25/min (1.4 keV) over a wide-angle range of 20–100 was used.

## 3. Results and Discussion

### 3.1. Thermal History

**Figure 2:**
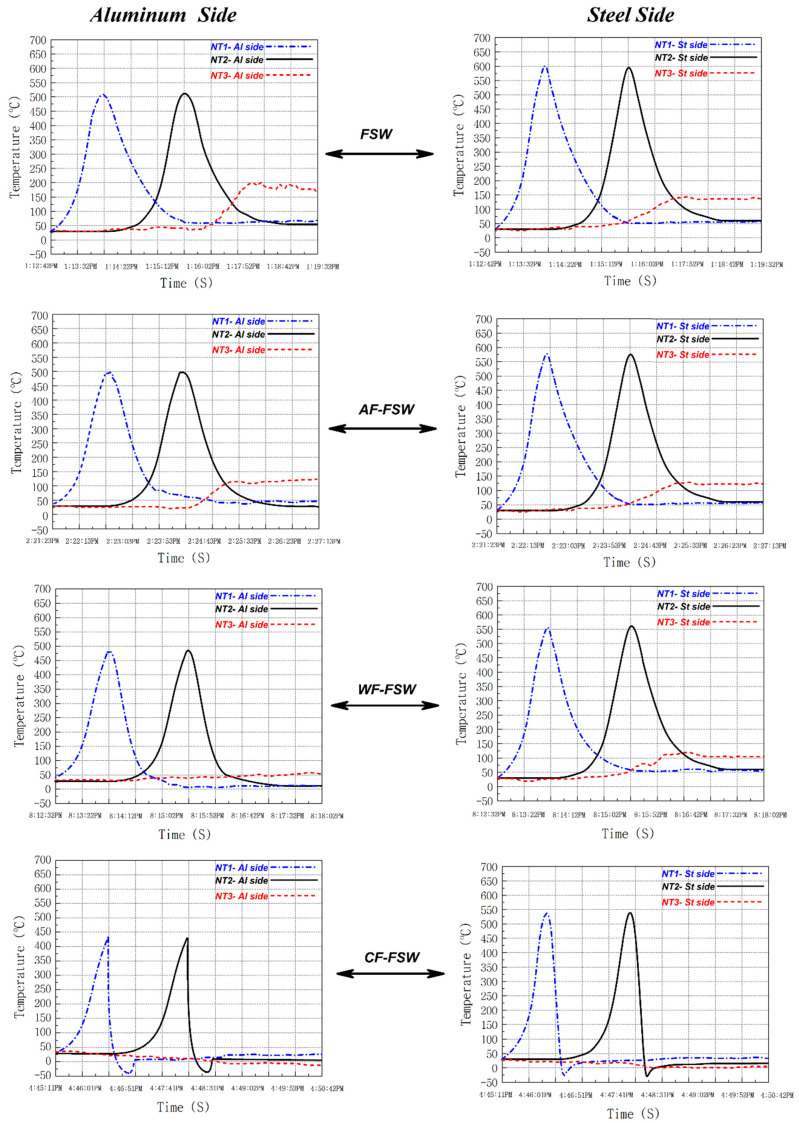
Thermal history of the workpiece according to the time on steel and aluminum side.

Thermal history is a very important parameter that alters the weld quality, second phase formation, and material flow. One of the main issues in the dissimilar FSW is the thermal, mechanical, and chemical properties difference of two metals like aluminum and steel or titanium. This causes defects at the weld zone such as wormhole, tunnel defect, or micro-cracks [27]. Figure 2 shows the thermal history of steel (advancing side) and aluminum (retreating side) during the welding process recorded by NT1, NT2, and NT3. The advancing side temperature is higher than the retreating side due to the higher viscosity of steel which forces the tool to apply a higher shear stress on the material increasing heat production in this part. On the other hand, the lower heat transfer coefficient of the steel caused a higher heat concentration in the steel side [41,42,43,44]. As it is obvious in the NT2 curves, the CF-FSW sample had higher cooling rate and the FSWed sample had lower cooling rate. This behavior happened due to the higher heat transfer between CO_2_ with base materials and lower heat transfer of air compare to other coolant. The effect of flow of coolant (air) was obviously detectable compared to immobility. The boiling point of CO_2_ is subzero at ambient condition; consequently, when the liquid CO_2_ sprinkled from the nozzle and dropped on the hot joint line, it absorbed its latent heat of evaporation cooling down the base metals. In the case of WF-FSW, the water is liquid until 100 °C and absorbs heat with specific heat capacity that is smaller than the latent heat of evaporation of CO_2_, thus the cooling rate and cooling capacity of CO_2_ is larger than water [45]. With AF-FSW, the specific heat capacity of the air is smaller than the specific heat capacity of water; consequently, the cooling rate and cooling capacity of water is larger than those of compressed air [46]. The recorded temperature by NT3 in both aluminum and steel side proved that the generated heat diffuses into the leading edge (LE) of the tool. In FSW, the heat diffusion was higher than in other cases and revealed that the preheated area prepares lower viscosity materials to flow from LE into the stir zone (SZ) [47]. In contrast, −35 °C of CO_2_ cooled materials increased their viscosity in LE. Higher material viscosity is one of the main reasons for the decrease of heat generation at CF-FSW case (~100 lower than FSW sample). This trend is predictable for other cases. Higher cooling rate changes materials’ viscosity (shear stress) on LE and changes total generated heat in SZ. This result was approved by NT3 thermocouple results. Due to the above discussion, in the case of the higher cooling capacity of liquid CO_2_, lower heat input influenced material flow and second phase formation, to be discussed in the next section. The cooling rate and cooling capacity can be enhanced by increasing the mass flow of cooling media and the rest of the cooling capacity depends on the nature of the cooling media and the energy needed for its phase transformation.

### 3.2. Material Flow

**Figure 3:**
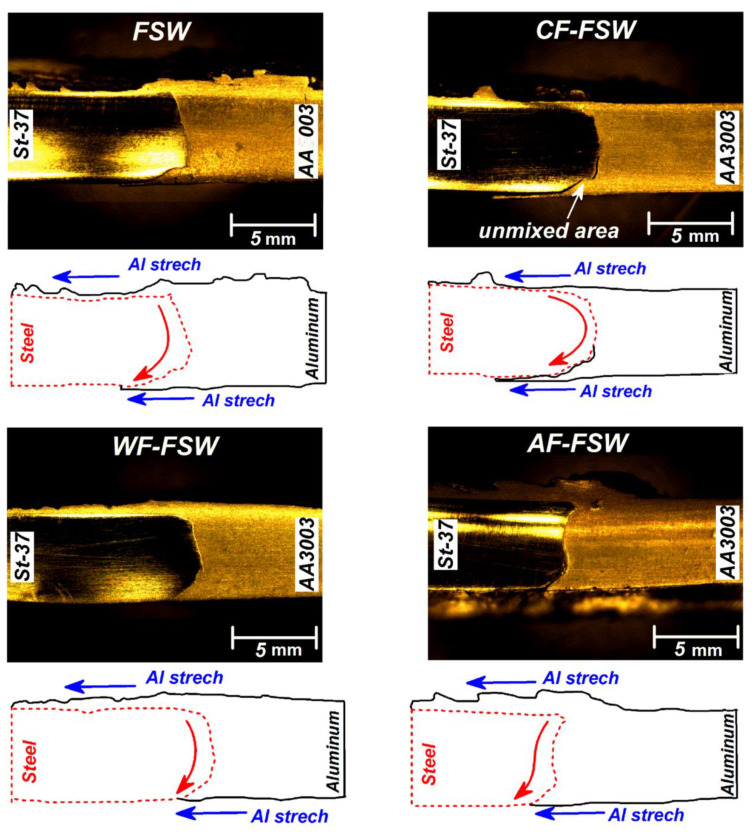
Weld cross-section macrograph of FSW, CO_2_-forced cooling FSW (CF-FSW), water-forced cooling FSW (WF-FSW), and air-forced cooling FSW (AF-FSW) samples and schematic view of internal material flow.

The overall material flow initiated from shoulder induced flow with stirring nature, then the downward and upward probe driven flow caused the vortex flow [48]. There are some issues in dissimilar joining via FSW due to mechanical, chemical, and thermal properties differences and these differences result in more severe plastic deformation in the aluminum side [49,50]. In fact, in dissimilar material joining using FSW, the appropriate material flow, weld morphology, and macrostructure shows a heterogeneous combination of two different metals. Too much difference between raw materials properties makes abnormal and insufficient material flow, especially at the stronger material side. Figure 3 shows the macrograph and schematic view of flow pattern in the welded joints in various cooling conditions. The conventional FSWed joint shows a smooth curvy interface between base metals and some stretch of steel in the aluminum side at the bottom of joint [34].

In fact, the raw materials interface was at the dagger-shaped area at the bottom of the joint, but higher softening of AA3003 during stirring action and stretching of steel into softened aluminum caused formation of this morphology [32,51,52,53]. Then, it can be seen that the base materials interface shifted into the aluminum side. This behavior can be seen in all samples. The CF-FSW shows crack formation at the weld root and bonding at the top of the weld due to the higher material flow and higher temperature at the top, while the temperature and flow are insufficient at the weld root, so, the root is the most critical area in this type of weld. Forasmuch as the CO_2_ cooling media causes higher cooling rate among other conditions, complete material flow is suppressed especially at the weld root and causes weak mechanical bonding. Additionally, excess cooling rate seems to lead to high contraction and separation of base metals [46]. The lower stirring action and weaker mechanical interlocking at lower area of stir zone are the main hypothesis for this material behavior. Stronger mixing and mechanical interlocking in the upper area prevents disintegration. The macroscopic observation of WF-FSW did not show any segregation or other defects. Due to the lower cooling rate than CO_2_ media, the higher heat concentration on the joint line, and more appropriate material flow, better mechanical bonding is achieved. In the cooling assisted processes, air-cooled samples have the lowest cooling rate and the most heat concentration after simple FSW. This caused the mixing pattern to be more similar to FSW joint. As mentioned before, the flowing air moderated heat concentration on the joint line and increased cooling rate slightly.

**Figure 4:**
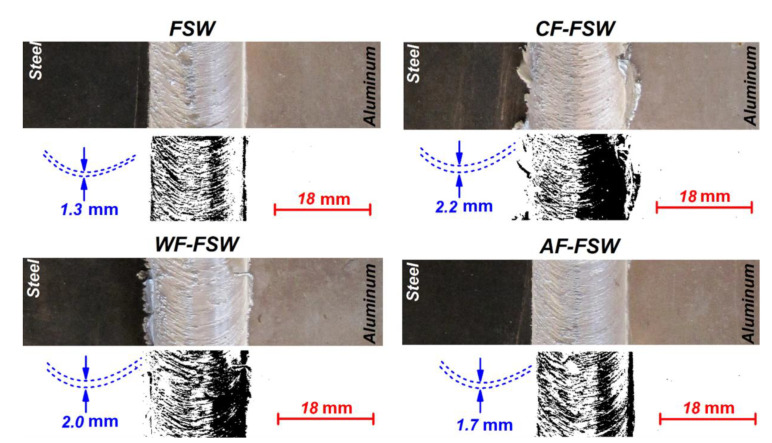
Surface analysis of various joints.

The surface flow of joints with flow analysis are depicted in Figure 4. As seen, the rough surface flow is formed in higher cooling samples. As expected, this behavior in the surface is affected by material viscosity. In all samples, the materials in direct contact with the shoulder experienced more thermo-mechanical cycling compared to other areas and the materials’ viscosity changed surface flow rings. The lower distance between flow rings formed in FSW and lower cooling cycle indicates a concentration of materials on the joint line. It seems the FSW tool pushes more stress in lower viscosity metals in (trailing edge) TE which leads to a higher mixing of dissimilar materials at the top of the joint.

### 3.3. Micro Structure

**Figure 5:**
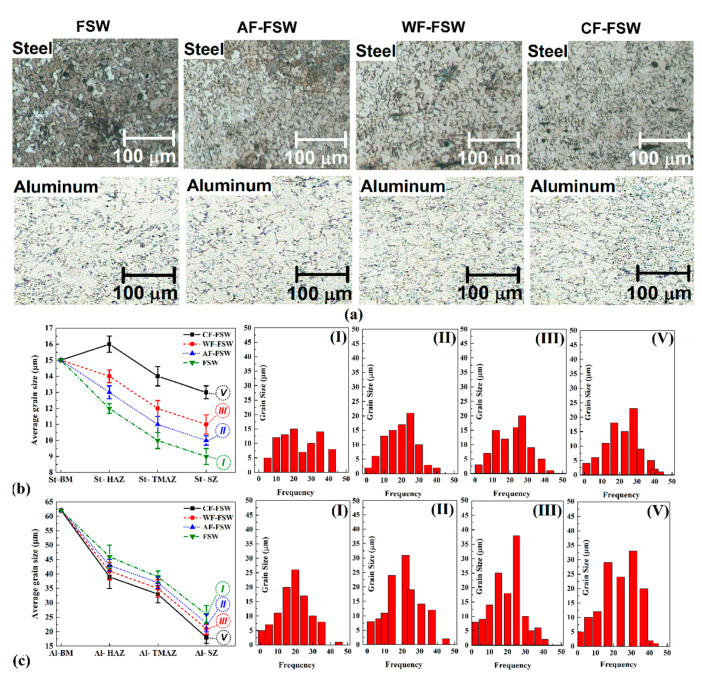
(**a**) Optical microscopy (OM) microstructure observations of various stir zone, (**b**) average grain size of steel side for different cooling conditions with histogram of grain size in stir zone (SZ) and (**c**) average grain size of aluminum side for different cooling conditions with histogram of grain size in SZ.

Figure 5a shows the optical microscopy (OM) image of SZ microstructure in different samples. All samples exhibit formation of equiaxed grains due to the dynamic recrystallization (DRX) phenomenon. With the study of the SZ, the changes in grain size at both sides are detectable. It seems that the cooling rate can affect DRX notably in the cooling stage, because as the cooling rate decreases, grain size increases. Grain size distribution is more inhomogeneous in the case of CF-FSW and can be attributed to the incomplete DRX mechanism or lower grain growth of nucleus which has been created on grain boundaries. The grain refinement intensity is higher on the aluminum side because the tool offset is toward the aluminum, causing severe deformation in the surroundings.

However, the average grain size is smaller in a higher cooling rate, so, as can be seen in Figure 5a, grain size decreases in the case of FSW, AF-FSW, WF-FSW, and CF-FSW, respectively. Figure 5b,c shows the average grain size of the steel and aluminum side in different samples and the histogram of grain size distribution at SZ of all samples. As it can be seen in Figure 5b, the grain size of base metal (BM) is 15 µm, and the grain size of steel is reduced in SZ with respect to the size of DRX. The grain size of steel increased in thermo-mechanical affected zone (TMAZ) in comparison with SZ due to lower deformation that TMAZ experienced. The heat affected zone (HAZ) of the steel side in the FSW sample has the highest grain size, even higher than BM grain size due to the static recrystallization that happened in this area [30,54]. As it was mentioned, the tool offset was toward the aluminum side and the weld line is far away from the steel side, so a higher fraction of the steel side is placed at the HAZ which experienced even higher temperatures without any deformation. Moreover, the lower thermal conductivity of steel in comparison with aluminum caused more grain growth on the steel side. When external cooling was employed in AF-FSW, WF-FSW, and CF-FSW, no HAZ was observed because the static recrystallization temperature did not occur due to the effect of the external cooling rate. In addition, lower working temperature caused the widening of TMAZ. Thus, the grain size of the HAZ of the steel side is smaller than BM. The prior mentioned fact is also valid for the aluminum side, and the high thermal conductivity of aluminum reduced the time at high temperature causing a significant grain growth. In addition, external cooling could help to inhibit static recrystallization and reducing working temperature. Figure 5c indicates that higher grain refinement occurs on the aluminum side which is attributed to higher deformation that is applied on the aluminum side via tool offset. It must be noted that the aluminum base metal grain size is 60 µm which is higher than that of steel(15 µm) and reaches to below 20 µm in CF-FSW. The results show that a higher cooling rate can reduce grain size due to suppressing completeness of grain growth kinetics as discussed in the whole of this section. Therefore, the grain size of each side decreases in the sample of AF-FSW, WF-FSW, and CF-FSW, ascendingly.

### 3.4. Intermetallic Compounds

**Figure 6:**
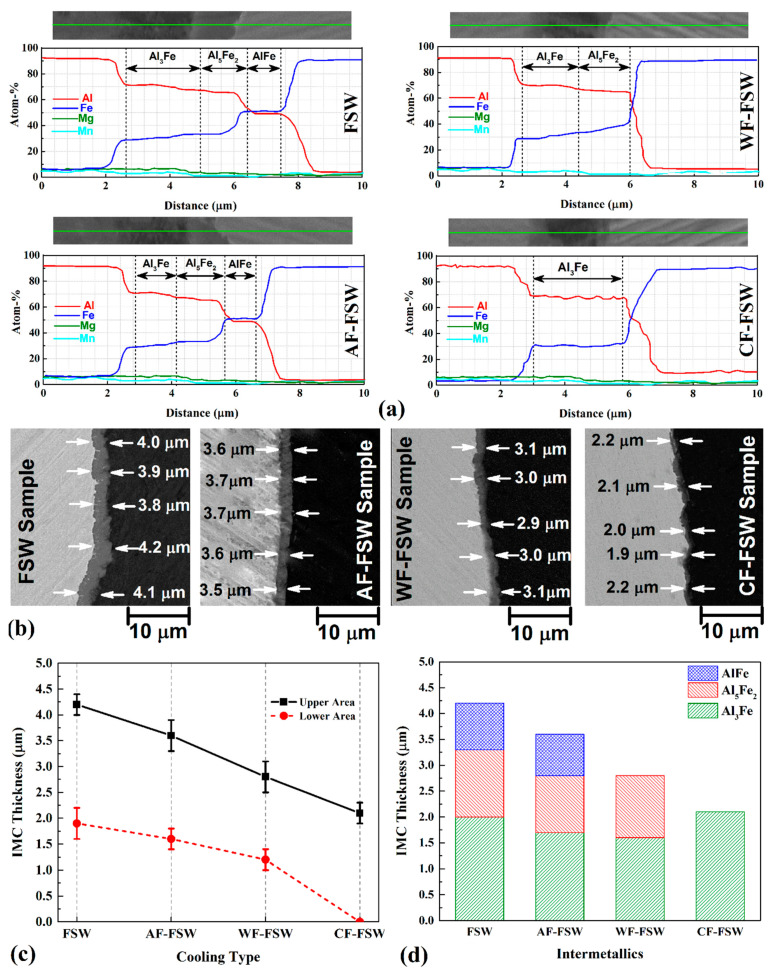
(**a**) EDS line scan of various samples, (**b**) SEM image from interface of joints, (**c**) intermetallic compounds (IMC) thickness in top and bottom of the weld, (**d**) thickness of each IMC for different cooling condition.

A vast variety of Al–St IMC with different morphologies can be formed during solidification of the Al–Fe system but the formation of Al–Fe system IMCs can occur in a solid-solid state by the solid-diffusion phenomenon [55]. In solid-solid diffusion for Al–St, the phase formation is followed by an order of priority of Al-richer phase formation, tightly. The activation of the Al–St system intermetallic increases when IMCs turn to Fe-rich type. The onset of formation temperature of the Fe-richer phase is higher than those of the Al-richer phase. Thus, the Al-richest phase occurs as Al_3_Fe(θ) is formed at first during warming up the specimens by diffusing Fe atoms to the aluminum substrate. Other more stable phases with a higher concentration of Fe atoms can be formed via diffusion of furthur Fe atoms to the previous IMC. Due to the recorded thermal history, there are chances to form Al_3_Fe(θ) and Al_5_Fe_2_(η), and FeAl, by raising the temperature or extending time at a lower temperature. Figure 6a shows the the line scan EDX spectra from the interface of Al–St joints. Results of chemical element percentage show that Al_3_Fe(θ) can be formed at first by diffusion of Fe atoms into the aluminum substrate and then by diffusing Fe atoms into Al_3_Fe(θ). Al_5_Fe_2_(η) can be formed with lower kinetic due to lower diffusion coefficient of Fe into Al_3_Fe(θ) at the same temperature. Al-richer IMCs seem to augment with increasing temperature or extending the time. Therefore, higher IMC thickness can be seen at lower cooling rates [56]. The longer time or lower cooling rate of FSW and AF-FSW exhibit thicker Al_3_Fe(θ) that is transformed into Al_5_Fe_2_(η) in order to pass the time by diffusing Fe atoms into Al_3_Fe(θ) at the interface of steel. Finally by further time in lower cooling rate, Al_5_Fe_2_(η) can be transformed to FeAl, from Fe-richer phase by diffusing of furthur Fe into Al_5_Fe_2_(η) at the interface of steel and IMC layer [39].

By passing time via lower cooling rate, the Al-rich phase as Al_3_Fe(θ) with lower activation energy, has more time of diffusing Fe to Al, and finally, further IMC thickness can be obtained by decreasing cooling rate. On the other hand, IMC growth is much greater for Al-richer phases with lower activation energy and higher diffusion temperature. Figure 6a proves the above-mentioned discussion where longer duration time at higher temperatures can cause IMC thickening and help to form Fe-richer IMCs like Al_5_Fe_2_(η) and FeAl. Fe-richer IMCs are formed by diffusing Fe atoms to previous IMC and start to grow from the interface of the steel side toward the aluminum side. As Fe concentration is higher, it is closer to the steel side. Thus, Al_3_Fe(θ) which is the Al-richest phase here, was formed at first close to the aluminum side, then Al_5_Fe_2_(η) was in the middle, and FeAl which is the Fe-richest phase is close to the steel side. Figure 6b shows interface of base metals at the upper area of the joint. The average thickness of IMC is marked for statistic information. The whole IMC layers thickness increased with a lower colling rate and it reached 4.25, 3.25, 2.75, and 2.25 µm for FSW, AF-FSW, WF-FSW, and CF-FSW, respectively (Figure 6c,d). In the FSW sample, the thickness of all IMC layers is much greater than the AF-FSW due to longer time duration at high temperatures. In WF-FSW and CF-FSW, the formation of the Al_5_Fe_2_(η) IMC layer is suppressed due to lower time duration or higher cooling rate. In the CF-FSW sample, even Al_5_Fe_2_(η) IMC layer formation is suppressed due to the highest cooling rate, and there is just a single phase IMC layer as Al_3_Fe(θ). Because of the lower activation energy of Al_3_Fe(θ), it can grow easily with a high constant rate, even at higher cooling rates. In the CF-FSW sample, duration time is not enough to form Al_5_Fe_2_(η) phase, thus, a thicker Al_3_Fe(θ) IMC layer is observed, because there is the non-transformed thickness to Al_5_Fe_2_(η) in comparison with other samples which exhibit Al_5_Fe_2_(η) phase. Based on previous works and obvious facts, the temperature under the shoulder region is higher than the temperature around the tool pin near the root. This is because of the higher frictional area of the shoulder due to the higher diameter, and the weld root is far from the main heat generation source. Thus, the higher temperatures and the bigger plasticisized area which is located on the top of the weld, lead to the formation of thicker and even more stable (Fe-richer) intermetallics, and various IMCs can be seen along the thickness of the weld. Therefore, temperature reduction along with the thickness toward the root cause lower IMC growth and thickening. In fact, the formation of Fe-richer phases is suppressed gradually toward the root in a way that only Al-richest IMC are formed at the weld root, or even no intermetallic exist. The IMC thickness decreases from the upper region of the weld through the weld root along with the thickness and the whole IMC thickness decrease since cooling rate increase. In the CF-FSW sample, there is no IMC layer at the weld root due to insufficient temperature and energy to form any IMC layer. Generally, lower temperature and duration time causes lower diffusion and even can suppress the formation of any IMC layer even Al_6_Fe non-equilibrium phase [55].

### 3.5. Tensile Strength and Fractography

**Figure 7:**
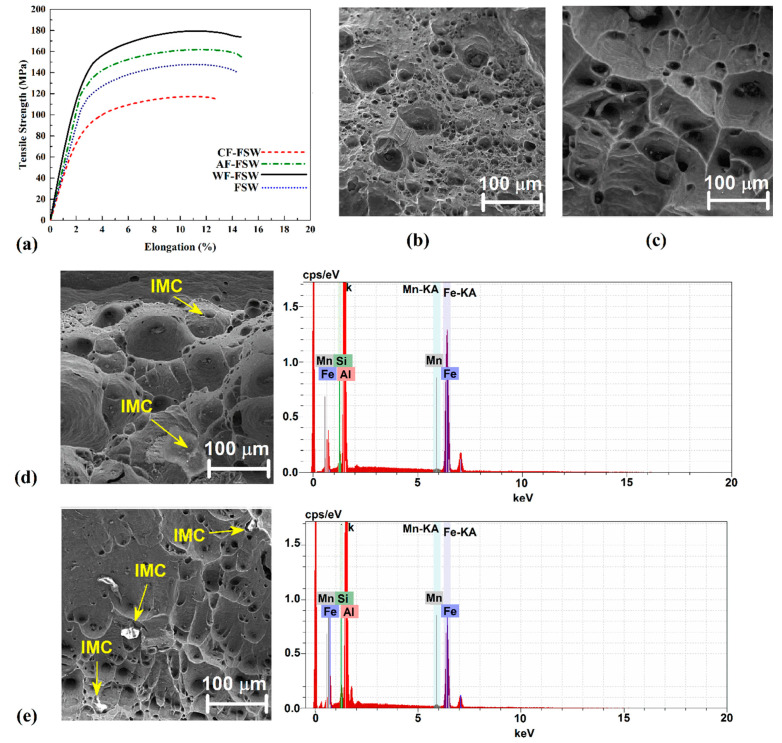
(**a**) Stress-strain curve of each cooling condition. SEM image of fracture surface of (**b**) CF-FSW and (**c**) WF-FSW samples. SEM image of fracture surface of (**d**) AF-FSW and (**e**) FSW sample with EDX result.

Figure 7a shows the stress-strain curve of all samples. It was noted that due to the significant difference of thermal expansion coefficient between steel and aluminum, there is the need for continuous metallurgical bonding with the lowest brittleness which is provided with a thin IMC layer [57,58,59]. However, the material flow and mechanical bonding are suppressed by a higher cooling rate so the CF-FSW sample exhibits the weakest mechanical bonding especially at the weld root that always is the critical part of the weld due to the weakest material flow and temperature here. Furthermore, higher cooling rate can cause more inhomogeneous shrinkage and distort the weld interface to cause segregation. Therefore, in the CF-FSW sample, root crack can be seen at the weld root due to no IMC formation and lack of proper mechanical bonding and higher distortion. In addition, it seems that discontinuous IMC layer formation at the interface with just the brittlest phase (Al_3_Fe) is the main reason for the lowest strength of the CF-FSW sample. Higher IMC thickening of samples leads to brittleness and causes lower tensile strength but it is higher than CF-FSW because of better mechanical bonding, lower distortion, and continuous IMC layer with lower Al-richest brittler phase. As seen, the AF-FSW sample has higher tensile strength than FSW that is related to a better material flow and mechanical bonding. It must be noted that macrocracks beside the microcracks and voids within the IMC layers decrease the weld strength dramatically. Results show that WF-FSW exhibits the highest tensile strength. IMC layer thickness of WF-FSW seems to be near the optimum value that is thick enough to exhibit lower brittleness. It is also continuous at the interface and the Fe-richer phase is formed by diffusing of Fe into Al_3_Fe(θ). It decreases the thickness of brittle θ phase and causes more ductility in comparison with CF-FSW. The material flow and mechanical bonding seem to be proper in the WF-FSW sample, so it can be concluded that IMC thickness and continuouity is the main controlling parameter for weld tensile strength besides proper mechanical bonding to stand shrinkages and distortions with lower brittleness, simultaneously.

Figure 7b shows the fracture surfaces of the tensile-tested specimens under SEM with secondary electrons to detect fracture topography. The CF-FSW sample fracture place was at the interface of the base metals (Figure 7b). SEM image from fracture surface exhibits brittle fracture with high cleavage pattern. Due to obtained results from material flow and metallurgical investigation, it seems that this fracture morphology is attributed to the lack of mechanical bonding and enough IMC formation at Al-St interface in CF-FSW joint. The WF-FSW sample shows dimples as well, but river pattern and some more defects like small voids can be seen which revealed semi-ductile fracture properties. It indicates a very proper mechanical bonding due to good material flow along the whole thickness of the WF-FSW sample. The fracture location on tensile sample was on HAZ of the aluminum side. For this reason, the fracture pattern is indicated in the aluminum side behavior. With a lower cooling rate in AF-FSW sample, IMC particles on the fracture surface appeared. The EDS point analysis of these particles indicated a mixture of Al–Fe–Mg–Si as the elements in parent metals. At the top of the picture, brittle fracture can be seen as a sign of more cleavage due to poorer mechanical bonding with more brittleness of the IMCs than the WF-FSW sample. The fracture location in this sample allocated to TMAZ area at aluminum side. The FSW sample exhibits larger dimples that show more ductile behavior due to the lower cooling rate of the FSW sample which does not suppress enough the grain growth besides the presence of some cleavages to show some brittle behavior. In addition, some IMCs can be seen in the FSW sample especially at the bottom of the picture due to the thickest IMC layer that plays an important role in crack initiation. The EDS analysis of IMC on fracture surface detected parent materials’ elements, as was comprehensively discussed before. In this case, the fracture location was on SZ after tensile test.

### 3.6. Hardness

**Figure 8:**
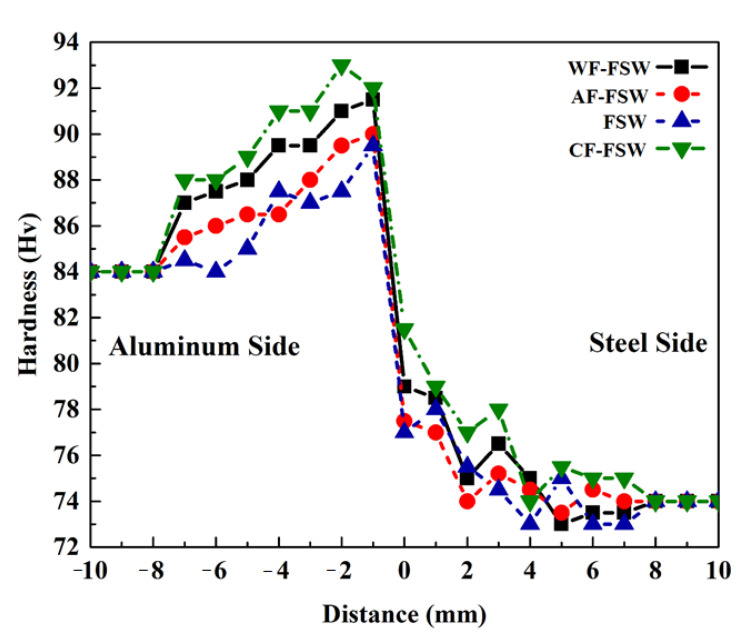
Hardness evaluation of each cooling condition according to distance.

The Vickers micro-hardness profiles across the thickness section of processed dissimilar FSWed different samples are shown in Figure 8. Aluminum base metal hardness is about 84 Hv and steel base metal hardness is about 74 Hv. Based on previous discussions, the hardness of SZ is the highest, and the HAZ hardness is the lowest if it exists. A sharp rise of hardness about −1 mm to −2 mm distance is attributed to IMC brittle phases that are composed at the interface of two dissimilar metals and are shifted right because of tool offset toward the aluminum side. The high hardness values of Al-richer agrees with literature [13,30,45,49,57]. As was mentioned in Section 3.2., the Al-richer phase is mostly formed with higher thickness individually in the CF-FSW sample that exhibits a maximum hardness of 93 Hv. The peak hardness of the CF-FSW sample is near to the aluminum side where the harder Al_3_Fe(θ) phase is with lower Fe content exist there. Other samples contain other ductile IMCs with different thicknesses with respect to the cooling rate which can change the kinetics of IMC formation and diffusing phenomena. In the lowest cooling rate, which is attributed to FSW and AF-FSW samples, respectively, even FeAl is formed as Fe-richest phase with higher ductility in present work. In the WF-FSW, the Al_5_Fe_2_(η) phase is observed as the most equilibrium phase with better ductility than Al_3_Fe(θ) and lower ductility than FeAl. In addition to all of the IMC observations, finer grains are achieved by a higher cooling rate that can lead to higher hardness. Based on all the above discussions, the CF-FSW sample exhibits higher hardness in comparison with other samples. The hardness reduction occurs in WF-FSW, AF-FSW, and FSW samples, descending, in a way that the lowest hardness peak is related to the FSW sample that is about 89 Hv. It must be noted that the peak hardness of AF-FSW and FSW samples is near to each other because of similar IMC layer composition but more hardness difference between CF-FSW and WF-FSW can be attributed to the Al_5_Fe_2_(η) phase which exists in the WF-FSW sample that caused hardness reduction. The hardness value decreased by distancing the weld line, so, the hardness of each side decreased in TMAZ and HAZ. The HAZ can be eliminated by a higher cooling rate too but a negligible decrease is seen for the FSW sample due to lowest cooling rate. At higher temperatures, the lower thermal conductivity of steel can produce a smaller HAZ as a hardness decrease can be seen around 6 to 8 distance in the FSW sample. Distances of −2 to −4 mm which exhibit constant hardness may belong to Al-side SZ with a value of 91, 89, 87, and 86 Hv for CF-FSW, WF-FSW, AF-FSW, and FSW, respectively.

## 4. Conclusions

In this research, dissimilar friction stirs welding of aluminum AA3003 and A441 AISI steel sheet have been done. The effect of various cooling media and their relation with the mechanical and metallurgical properties of joints are considered and the final results presented as follows:

The thermal history of each side indicates that peak temperature is constant but the cooling rate is directly attributed to the cooling capacity of each medium and CF-FSW exhibits the highest cooling rate among selected cooling media. Microstructural observations show grain refinement via dynamic recrystallization mechanism (DRX) at the nugget zone (NZ). A higher cooling rate causes grain growth suppression during DRX to form smaller grains.

Intermetallic compounds analysis indicated that various IMCs can be formed in different cooling conditions. Al-richer phases are formed firstly by lower activation energy. Fe-richer phases are formed via further Fe atoms diffusing into previous IMCs with a lower diffusion rate. IMC layers in the FSW sample consist of AlFe as a Fe-richest phase, WF-FSW sample IMCs consist of Al_3_Fe(θ) and Al_5_Fe_2_(η) phases, and CF-FSW sample single IMC layer consists of Al_3_Fe(θ).

The WF-FSW sample has the highest strength due to the optimum thickness of IMC layers to form metallurgical bonding with lower brittleness beside proper material flow to form mechanical bonding. A lower cooling rate allows thickening of IMCs and provides more brittleness to decrease the strength of FSW and AF-FSW, respectively. FSW exhibits more strength simply due to better mechanical bonding and lower distortion than AF-FSW. The lowest tensile strength was related to the CF-FSW sample due to single brittle Al-richer phase formation, lack of IMC formation at the weld root, lack of proper material flow which causes poor mechanical bonding at the weld root, and higher distortion.

Hardness evaluation indicates that a higher cooling rate provides higher hardness due to the limitation of grain growth during dynamic recrystallization as well as inhabitation of Fe-richer phase formation which has a lower hardness. The hardness increase of the aluminum side is stronger due to higher deformation which is applied on the aluminum side because of tool offsetting toward the aluminum side.

## Figures and Tables

**Table 1:** Mechanical properties of base metals.

Parameters	A441 AISI Steel	AA3003 Aluminum Alloy
Density, kg/m^3^	7800	2730
Melting Point, °C	1400	644
Thermal conductivity, W/m·k	42.7	154
Specific heat capacity, J/g-°C	0.477	0.893
Yielding strength, MPa	344	186
Ultimate tensile strength, MPa	580	200
Shear strength, MPa	380	110
Elongation, %	15	10
Vickers hardness	355	90

**Table 2:** Chemical composition of base metals.

**AA3003 Aluminum Alloy**						
Al	Fe	Si	Zn	Mn	Cu	
97.1	0.7	0.6	0.2	1.2	0.20	
**A441 AISI Steel**						
S	P	C	Mn	Cu	Si	Fe
0.05	0.04	0.22	1.00	0.20	0.40	97.00

**Table 3:** Friction stir welding (FSW) process parameters.

Parameter	Tool Rotational Speed	Tool Traveling Speed	Tool Tilt Angle	Tool Plunge Depth	Tool Offset
Value	800 rpm	40 mm/min	2	0.2 mm	1.3 mm in Al side

## Data Availability

Data is contained within the article.
